# Research progress of assessment tools for Parkinson’s disease psychosis in China

**DOI:** 10.3389/fpsyt.2025.1588618

**Published:** 2025-07-10

**Authors:** Shuchan Wang, Baoqin Yang, Ping Lu, Yaping Chen, Jianing Dai, Lijun Zhao, Miaomiao Wang, Jiahuan Yu, Lixiu Zhang

**Affiliations:** ^1^ Nursing Department, The Affiliated Kangning Hospital of Ningbo University, Ningbo, China; ^2^ Medical College, Huzhou University, Huzhou, China

**Keywords:** Parkinson’s disease, psychosis, Parkinson’s disease psychosis, assessment tools, review

## Abstract

Parkinson’s disease psychosis (PDP) has a significant impact on the quality of life and disease prognosis of patients with Parkinson’s disease (PD). Accurate assessment of psychotic symptoms in PD patients is critical for early detection and intervention. This, in turn, improves clinical outcomes and quality of life. This review evaluates both internationally validated and China-specific assessment tools for PDP, including the Parkinson’s Psychosis Questionnaire, Geriatric Apathy Scale, and culturally adapted instruments. We analyze the psychometric properties, cultural applicability, and clinical utility of these tools in Chinese populations. Our findings provide evidence-based recommendations for healthcare professionals to optimize tool selection, enhance diagnostic accuracy, and guide targeted interventions in China’s diverse clinical settings. The review also identifies gaps in current assessment approaches and proposes directions for future tool development and validation studies.

## 1 Introduction

Parkinson’s disease psychosis (PDP) is a common non-motor symptom of Parkinson’s disease (PD), with risk factors including advanced age, prolonged duration of the disease, disturbances of sleep, and exposure to medications (1). PDP manifests itself through a spectrum of positive symptoms, such as passage, pseudohallucinations, hallucinations, and delusions (2). Psychiatric symptoms in patients with PD often exhibit sequential progression (3, 4). In the early stages of the disease, PDP symptoms predominantly include minor phenomena such as illusions, pseudo-hallucinations, and passage (5, 6). Minor phenomena are early symptoms of loss of insight or paranoid delusions in patients with PD. Among these, visual illusions are the most common manifestation (7), characterized by the misidentification of objects by the patient.

Furthermore, Schneider et al. (5) conducted a survey involving 5,950 patients with PD, revealing that approximately 10.2% of the patients experienced pseudo-hallucinations, while 37.8% exhibited the symptom of “passage”. This symptom is characterized by sudden freezing or difficulty in movement as patients with PD navigate through doorframes, narrow spaces, or crowds. In advanced stages of the disease, patients were likely to develop hallucinations such as visual, auditory, olfactory, and tactile hallucinations and delusional symptoms. Visual hallucinations are the most common psychotic symptoms in patients with PD, with an incidence rate of 22% to 43%, occasionally accompanied by olfactory or auditory hallucinations (8). In clinical practice, visual hallucinations are often among the symptoms most readily recognized by patients, frequently prompting them to seek medical attention. In addition, these symptoms can help to distinguish PD from other types of Parkinsonian syndrome (8). A meta-analysis (9) reported that the prevalence rates of auditory, olfactory, and tactile hallucinations in patients with PD were 7.1%, 3.3%, and 2.6%, respectively. Furthermore, studies indicate that the prevalence of delusions in PD ranges from 5% to 17.9%, representing one of the most severe psychotic manifestations (10). These symptoms may lead to adverse outcomes, including violent behaviors and increased suicide risk. Delusions in patients with PD can be classified as isolated delusions, paranoid delusions, persecutory delusions, and jealousy-related delusions. Among these, paranoid delusions are the most common, accounting for approximately 82.6% of delusional cases in patients with PD (10). Despite growing research on PDP, public awareness remains limited. This highlights the need for improved dissemination. International scholars have developed various PDP screening tools, some of which have undergone multiple iterations and have been validated and promoted globally. Nevertheless, their usability and diagnostic accuracy still require improvement. Meanwhile, the application and development of assessment tools in China remain in the exploratory stages. Therefore, this review aims to provide an overview of the concept and assessment tools for PDP to enhance awareness, facilitate early detection of PDP symptoms, and offer a reference framework for developing intervention strategies.

## 2 The impact of psychosis

The impact of psychosis According to epidemiological data (11), the incidence of PDP is approximately 4.28 cases per 100 person-years. Neuropsychiatric symptoms are indicative of disease progression in PD and are significantly associated with increased rates of hospitalization and mortality (12). Studies have shown that PD patients experiencing hallucinations exhibit a more rapid decline in cognitive function(?) and a 71% increase in all-cause mortality (11). Early neuropsychiatric symptoms often precede overt cognitive impairment and may serve as important predictors of the transition from PD to dementia (13). PDP can lead to impaired reality testing and social dysfunction, severely compromising sleep and overall quality of life. These symptoms also place a substantial burden on caregivers, particularly due to nocturnal behavioral disturbances and aggressive behaviors. (14). Furthermore, impulse control disorders, such as pathological gambling and binge eating, may result in devastating financial consequences for affected families (15).

## 3 Assessment tools for psychotic disorders in Parkinson’s disease

### 3.1 Screening-oriented tools

#### 3.1.1 The Movement Disorder Society Unified Parkinson’s Disease Rating Scale

The MDS-UPDRS was developed by Goetz et al. (16) and later translated into Chinese by Yan Qu et al. (17). It is currently the most widely used scale for assessing motor and non-motor symptoms in patients with PD. Evaluations are performed by professionals based on reports from patients or their caregivers. The hallucinations and psychosis subsection (item 1.2) of Part I can be used for the preliminary screening of PDP, focusing on behaviors over the past week. This item is rated on a 0 to 4 scale, where higher scores indicate more severe symptoms. The Cronbach’s alpha for Part I is 0.79, indicating good reliability and validity (16). This scale features simple and user-friendly items, making it widely used in clinical practice and facilitating the timely identification of psychiatric abnormalities in patients with PD for early intervention. However, its assessment of psychiatric symptoms remains relatively general, lacking specificity in evaluating the type and frequency of symptoms such as hallucinations and delusions. Consequently, it has low screening sensitivity and is not considered the most sensitive tool for detecting PDP (18).

#### 3.1.2 Parkinson’s Psychosis Questionnaire

The PPQ was developed by Brandstaedter et al. in 2005 (19) for the early assessment of drug-induced psychosis in patients with PD over the past month. The PPQ quantifies the frequency and severity of four clinical categories: sleep disturbances, hallucinations/illusions, delusions, and disorientation. It consists of 14 items across 4 dimensions. The scoring is based on a cross-rating of frequency and severity, with a Cronbach’s alpha of 0.680. The questionnaire has been translated into several languages, including Portuguese, but has not yet been adapted into Chinese. The PPQ is straightforward, with clear and specific items, and takes only 7 minutes to complete. It is suitable for quick assessments and is highly sensitive to drug-induced psychiatric disorders in early-stage PD. It is particularly useful for caregivers (20). However, the PPQ is not intended to replace comprehensive clinical evaluations for the final diagnosis of drug-induced psychosis. Its utility in the follow-up monitoring and intervention of patients with PDP still requires further validation.

#### 3.1.3 Scale for Evaluation of Neuropsychiatric Disorders in Parkinson’s Disease

The SEND-PD was developed by Spanish scholars Martinez et al. in 2012 to assess the presence and severity of neuropsychiatric symptoms (21). A study by Mayela et al. found that this scale is equivalent to the MDS-UPDRS in evaluating hallucinations, psychosis, emotional blunting, and other symptoms (22). The SEND-PD scale consists of three dimensions: psychiatric symptoms, emotional blunting/apathy, and impulse control disorders, with a total of 12 items, and takes approximately 5–10 minutes to complete. Severity is rated on a 5-point Likert scale. The Cronbach’s alpha for psychiatric symptoms, emotional blunting, and impulse control disorders are 0.730, 0.820, and 0.520, respectively. Although this scale has been adapted for Spanish, German, and other languages, its translation into Chinese remains an unmet need (23). Eichel et al. conducted a study using the SEND-PD to assess the impact of neuropsychiatric symptoms on health-related quality of life (HR-QoL) in PD patients and their caregivers. The study found a significant correlation between the emotional/apathy dimension of the SEND-PD and reduced HR-QoL in PD patients (p¡0.001). This suggests that the SEND-PD provides a valuable tool for quickly assessing neuropsychiatric symptoms in PD patients. With its few items and straightforward structure, the SEND-PD is easy for patients to understand and can be used for preliminary screening of psychiatric symptoms in advanced or demented PD patients. However, since the scale relies on information provided by caregivers, the assessment may be influenced by the caregivers’ subjective biases.

#### 3.1.4 Psychosis and Hallucination Questionnaire

The PsycH-Q was developed by Australian scholars Shine et al. in 2015 (24). It is designed for assessing PD patients without cognitive impairment and is an easy-to-use, effective,and self-administered tool. The PsycH-Q accurately identifies hallucinations and psychotic symptoms in PD patients and also evaluates their insight and the closeness between patients and their caregivers. The questionnaire consists of 5 dimensions and 20 items, with an assessment time of 5 to 10 minutes. Symptoms are scored on a 0 to 4 scale for both frequency and severity. The total score is calculated by summing the frequency scores of each item, with a possible range of 0 to 80. Frequency and severity scores can be calculated separately or combined (frequency × severity). The PsycH-Q demonstrates excellent internal consistency (Cronbach’s alpha = 0.900) and can be completed in under 10 minutes. This scale is highly sensitive to the frequency and severity of hallucinations and psychotic symptoms in PD patients, providing an effective tool for clinical treatment and disease monitoring. However, it should be noted that the PsycH-Q focuses primarily on hallucinations and lacks coverage of related symptoms such as delusions. As a self-report scale, it may be subject to underestimation or overestimation by patients themselves. Additionally, as the scale was developed recently, it has not yet been applied in China, and further large-scale validation studies are needed to confirm its reliability and applicability.

#### 3.1.5 Geriatric Apathy Scale

The GAS developed and validated by Yi H Jin 2024, is a culturally tailored instrument designed to assess multidimensional apathy in neurodegenerative disorders among Chinese populations (25). It demonstrates good internal consistency (Cronbach’s alpha = 0.862) and test-retest reliability (r = 0.767). The scale comprises 16 items across three domains: cognitive-social motivation, emotional response and expression, and behavioral initiation. Each item is rated on a four-point Likert scale based on experiences over the past month, with higher scores indicating greater apathy. The GAS offers several advantages: (1) cultural specificity for Chinese patients, (2) strong psychometric properties, (3) effective discrimination between apathy and depression, and (4) clinical cut-off for PD patients (15.5 points; sensitivity: 78.9%, specificity: 69.3%), facilitating rapid screening within 10–15 minutes. However, limitations include: (1) focus on apathy rather than core psychotic symptoms of PDP (e.g., hallucinations, delusions), (2) reliance on self-report, potentially biased in cognitively impaired patients, (3) lack of a caregiver-rated version, (4) limited cross-cultural applicability, and (5) absence of independent validation in PD populations and small sample size (n = 113), without consideration of motor subtypes. In summary, GAS is a reliable and efficient tool for assessing apathy in Chinese PD/PDP patients, particularly in capturing culturally influenced motivational deficits. However, comprehensive psychiatric evaluation requires complementary tools that target psychosis and behavioral symptoms.

#### 3.1.6 University of Miami Parkinson’s Disease Hallucinations Questionnaire

Papapetropoulos et al. developed the UM-PDHQ, a clinician-administered tool for healthcare professionals (18). The questionnaire consists of 6 quantitative items and 14 qualitative items, totaling 20 questions. The qualitative items are not scored. The UM-PDHQ is an effective screening tool for detecting hallucinations in PD patients, with the primary aim of drawing attention to the presence of hallucinations. However, it does not provide an overall severity score, nor is it a grading or rating scale. Additionally, the questionnaire is still in the early stages of development, having only preliminarily assessed the presence and characteristics of hallucinations in PD patients. Currently, it is only used in the Miami University area. Its reliability, validity, and effectiveness have not been systematically evaluated, and its broader applicability remains uncertain.

#### 3.1.7 Tottori University Hallucination Rating Scale

The TUHARS was developed by Japanese scholars in 2008 as a structured clinical interview (26). The scale evaluates five aspects: types of hallucinations, frequency, severity, caregiver burden, and nighttime psychiatric symptoms. It uses a Likert 4-point scale to assess frequency, severity, and caregiver burden. The types of hallucinations evaluated include visual, auditory, olfactory, tactile, and general hallucinations, each rated 1 point, with a total score accumulated (0 points if absent). The Cronbach’s alpha for the scale is 0.880. Japanese scholar Hirayama et al. used TUHARS to quantify hallucinations and assess the severity of PD in patients (27). They found a significant correlation between urinary 8-hydroxydeoxyguanosine levels and hallucinations, confirming that the scale provides a comprehensive and detailed assessment of hallucination symptoms in PD patients. This scale is concise, with an average completion time of 5 minutes, and is suitable for preliminary screening of hallucinations in PD patients. However, its focus is limited to hallucinations, and it has been less widely applied. Additionally, no Chinese version is available, and further validation of its reliability and validity is needed.

### 3.2 Diagnostic-oriented tools

#### 3.2.1 Positive and Negative Syndrome Scale

The PANSS (28) is a standardized assessment tool originally developed by Kay et al. and later translated into Chinese by Yanling He et al. (29). Initially designed to assess the severity of various types of symptoms in schizophrenia, Chinese and international research has confirmed that the PANSS also demonstrates robust reliability and validity in patients with PD. Furthermore, it presents a thorough assessment of the psychiatric syndrome in patients with PD (30). In 2000, Lancon (31) reclassified the PANSS into five dimensions: negative, positive, excitement, depression, and cognitive. In patients with PDP, the positive dimension is commonly used to gauge the severity of the syndrome. This dimension consists of seven items, including delusions, hostility, and hallucinations, each scored on a scale of 1 to 7, with a total score ranging from 7 to 49. The PANSS is primarily suitable for cognitively intact PD patients or when they are the sole source of information. It should be administered by a trained psychiatrist who evaluates the patient’s syndromal symptoms over the previous week. Completing the positive subscale typically takes about 7 to 15 minutes. The PANSS has been translated into several languages, including French and Arabic. The Cronbach’s alpha for the PANSS ranges from 0.730 to 0.830. A randomized controlled trial (32) involving 40 patients found that decreased metabolite levels in specific brain regions were associated with psychiatric symptoms in PD, as assessed by the PANSS. Wang et al. employed the PANSS to evaluate the feasibility of unilateral anterior capsulotomy combined with subthalamic nucleus deep brain stimulation (DBS) in advanced PD patients with psychosis (30). Their findings indicated that this approach could positively improve the psychiatric syndrome in PDP, offering an effective treatment option. Each PANSS item features a clear definition and specific operational scoring criteria, which facilitate comprehension and application by healthcare professionals. The scale is well-established, widely utilized, and regarded as a primary outcome measure for PDP (33). However, many items require evaluation by trained professionals, limiting the feasibility under limited human resources. In addition, the categorical evaluations of the scale do not differentiate among the various types of hallucinations or delusions, imposing certain restrictions on its precision in the clinical evaluations of patients with PDP.

#### 3.2.2 Brief Psychiatric Rating Scale

The BPRS was developed by Overall et al. in 1962 (34). Initially intended to assess the psychiatric symptoms of patients with schizophrenia over the past week through direct observation, it has recently been applied to patients with PD. The scale is observer-rated and covers five dimensions (including hostility and suspiciousness) as well as 18 items (including delusions). Each item is scored on a scale of 1 to 7, producing a total score ranging from 18 to 126. The scoring considers the intensity, frequency, duration, and functional impact of the symptom. A Chinese translation by Mingyuan Zhang et al. in 1984 yielded a Cronbach’s alpha of 0.871 (35). In a randomized controlled trial involving 21 PD patients, Stuebner et al. found that those with non-dipping nocturnal hypertension exhibited more severe psychiatric symptoms than those with dipping nocturnal hypertension (36). Using the Chinese version of the BPRS to evaluate neuropsychiatric symptoms in 209 PD patients, Dongdong Wu et al. confirmed its good applicability and reliability in this population (37). Clinically, the BPRS is widely used, reliable, and valid, focusing on the severity of psychotic symptoms in patients with PDP. It is considered a primary outcome measure for PDP (33), especially suitable for acute presentations of prominent positive symptoms. However, it requires 15 to 30 minutes to complete and does not differentiate specific types of hallucinations or delusions. This limitation restricts its accuracy in characterizing the exact symptom profile of PDP.

#### 3.2.3 Scale for the Assessment of Positive Symptoms for Parkinson’s Disease

Voss et al. simplified and adapted the SAPS-PD (38) based on the Scale for the Assessment of Positive Symptoms. It is a structured clinical interview that consists of two sections: hallucinations and delusions, with a total of 9 items. The scale evaluates visual, auditory, and tactile hallucinations, as well as persecutory delusions, jealous delusions, and illusions. A Likert 6-point scale is used, ranging from 0 to 5, indicating “none” to “severe or frequent,” and the assessment typically takes more than 30 minutes. Cummings et al. (39) used SAPS-PD in a 6-week randomized controlled trial with pimavanserin, validating its reliability in assessing psychiatric symptoms in PDP patients. With fewer items and a high level of familiarity, the SAPS-PD has high sensitivity for detecting minor phenomena, making it a recommended tool for assessing PDP (33). However, it lacks full psychometric validation and requires evaluators with a high level of professional expertise. The low degree of standardization limits its widespread adoption.

#### 3.2.4 enhanced Scale for the Assessment of Positive Symptoms in PD

In 2018, Kulick et al. (40)developed the eSAPS-PD by incorporating olfactory hallucinations, gustatory hallucinations, and minor phenomena into the original SAPS-PD to improve the identification of psychotic symptoms. The assessment time for eSAPS-PD is approximately 2 minutes for patients without psychiatric symptoms and over 10 minutes for patients with complex symptoms. Zhang Yu et al. (6) applied eSAPS-PD to 149 Chinese patients with PD, showing robust psychometric properties. The eSAPS-PD demonstrated a sensitivity of 28% for detecting psychotic symptoms in patients with PD. This was notably higher than the combined detection rate (11%) of conventional clinical assessments such as clinician impression, the MDS-UPDRS Part 1A. The eSAPS-PD was particularly effective in identifying minor hallucinations and less commonly assessed symptoms, including olfactory and gustatory hallucinations, which are often overlooked by standard tools. It demonstrates good applicability in cognitively impaired patients and strong utility in clinical decision-making.

#### 3.2.5 Parkinson’s Disease Psychiatric Rating Scale

The PPRS was developed by Friedberg et al. in 1998 (41) as a specialized tool for assessing psychiatric symptoms in patients with PD. It consists of 6 items, including visual hallucinations and persecutory delusions, along with one global assessment item. A 5-point Likert scale is used, ranging from 1 to 4, with a total score ranging from 6 to 24. The scale takes 5 to 10 minutes to complete, and its Cronbach’s alpha is between 0.760 and 0.800. In 2007, Visser et al. (42) revised the Parkinson’s Disease-Psychiatric Complications Outcome Scale (SCOPA-PC) based on the PPRS. Van et al. (43) used SCOPA-PC to assess the severity and progression of psychiatric symptoms in 396 PD patients with different motor subtypes and found that prominent gait instability and postural difficulty were associated with lower SCOPA-PC scores. The PPRS is simple to administer, requires little time, and allows for monitoring symptoms over time. Its visual hallucination item evaluates both the frequency of hallucination events and the patient’s insight during these occurrences. The scale has been widely used in PD patients in countries such as the United States, Canada, and Spain, and is considered reliable. It is suitable for assessing psychiatric symptoms and treatment effects in diagnosed PD patients. The PPRS fails to capture the full heterogeneity of PDP. Its limited scoring range also hinders effective tracking of symptom progression. Furthermore, only three items evaluate psychiatric symptoms, while the remaining items focus on related characteristics such as “confusion,” “sexual preoccupation,” and “sleep disturbances,” which are not strictly psychiatric symptoms. As a result, the final score may be influenced by non-psychiatric factors. Additionally, the scoring system groups multiple characteristics together (e.g., “3 points = frequent occurrence; lack of full insight; persuadable”) and does not assess hallucinations that occur frequently but with preserved insight. For these reasons, the PPRS is not suitable for screening for PDP (44), and it has not yet been adapted or validated for use in Chinese populations.

### 3.3 Composite tools

#### 3.3.1 Neuropsychiatric Inventory Questionnaire

The NPI-Q was adapted by Cummings et al. in 1997 from the Neuropsychiatric Inventory (45). It is an informant-based measure that relies on input from the primary caregiver of the patient. Originally intended to assess common neuropsychiatric symptoms in individuals with dementia, it has since been applied to patients with PDP. In 2010, Wanxin Ma et al. translated the instrument into Chinese and applied it to older patients with dementia in China (46). Its Cronbach’s alpha was 0.851, and its test-retest reliability was 0.860. In a cross-sectional study of 450 patients with PD, Chahine et al. (47) found that psychotic symptoms in these patients had a significant impact on caregiver burden. Yuan Fang (48) applied the Chinese version of the NPI-Q to 63 patients with PD who exhibited psychiatric symptoms, demonstrating strong reliability and validity. The scale assesses the patient’s mental and behavioral status over the previous month. It focuses on three dimensions: psychotic symptoms, emotional disturbances, and frontal lobe function, including 12 items of symptoms. The NPI-Q is divided into a severity subscale and a caregiver distress subscale. For the severity subscale, each item is first evaluated for the presence or absence of symptoms. If present, symptoms are scored from 1 to 3. Caregiver distress is rated from 0 to 5, independent of the severity score. The total NPI-Q score is the sum of severity scores for all 12 elements, ranging from 0 to 36. Administration takes approximately 10 to 15 minutes, and each item includes an explanation. The scale considers the caregiver’s perspective and is appropriate for the clinical evaluation of patients with PDP with cognitive impairment. However, its applicability to patients with PDP with severe psychiatric symptoms requires further examination. In addition, informants complete the scale independently, which may introduce bias due to factors such as cultural background, comprehension, and educational level. In addition, NPI-Q only provides a basic assessment of the presence and severity of PDP and does not classify specific subtypes of symptoms.

#### 3.3.2 The Movement Disorder Society Non-Motor Symptoms Rating Scale

The MDS-NMSS was developed by Chaudhuri et al. in 2020 (49), based on the Non-Motor Symptoms Scale for Parkinson’s Disease (NMSS), to evaluate symptoms over the past month. The scale includes 13 dimensions with 52 items, covering areas such as psychosis. The psychosis section consists of four items that assess symptoms such as illusions, hallucinations, and delusions. Each item is rated for frequency and severity on a 0 to 4 scale, with the total score calculated as the sum of the products of frequency and severity for all items. The Cronbach’s alpha of the scale is 0.660. The MDS-NMS is primarily used to evaluate PDP patients with mild to moderate psychiatric disorders or mild cognitive impairment. The scale features consistent scoring standards and provides detailed descriptions of each symptom’s clinical presentation, making it easy for researchers to understand and apply. However, the large number of dimensions, which include not only psychiatric symptoms but also other non-motor symptoms, may lead to mixed results. Additionally, the scale lacks sensitivity in assessing changes due to disease progression or treatment interventions and does not provide detailed evaluations of specific types of hallucinations or delusions. Further validation, including linguistic and psychometric testing, is needed.

## 4 Comparative analysis of assessment tools for psychiatric disorders in Parkinson’s disease

### 4.1 Comparison of basic information

A comparison of PDP assessment tools in terms of development time, assessment duration, and content framework is presented in **Table 1**. There is considerable variability in the symptoms and severity of PDP across different individuals and disease stages. Therefore, when selecting an assessment tool for PDP, it is important to choose one that is targeted and suitable. The objectives, assessment methods, scope of applicability, and assessment duration of different PDP tools vary.

**Table 1:** Comparison of basic information across different Parkinson’s disease psychosis assessment tools.

Assessment Tool	Development Year	Country	Assessment Duration	Content Framework	Cronbach’s alpha	Translation/Adaptation
MDS-UPDRS Part 1A (16)	2008	USA	5 min	6 items	0.734	Chinese, Italian, Hungarian, etc.
PPO (19)	2005	Germany	7 min	4 dimensions, 14 items	0.680	English, Portuguese, etc.
SEND-PD (21)	2012	Spain	5–10 min	3 dimensions, 12 items	Psychotic symptoms: 0.730, Emotional/Blunted: 0.820, Impulse control: 0.520	German, English, etc.
PsycH-Q (24)	2015	Australia	5–10 min	2 dimensions, 20 items	0.900	None
GAS (25)	2024	China	10–15 min	3 dimensions, 16 items	0.862	None
UM-PDHO (18)	2008	USA	10–15 min	2 dimensions, 20 items	Not applicable	None
TUHARS (26)	2008	Japan	5 min	5 dimensions, 7 items	0.880	English, etc.
PANSS (28)	1987	USA	7–15 min	5 dimensions, 33 items	0.730-0.830	Chinese, Korean, Japanese, French, etc.
BPRS (34)	1962	USA	15–30 min	5 dimensions, 18 items	0.871	Chinese, Italian, Portuguese, etc.
SAPS-PD (38)	2013	USA	more than 30 min	2 dimensions, 9 items	Not applicable	Chinese etc.
eSAPS-PD (40)	2018	USA	Without psychiatric symptoms: 2 min; With psychiatric symptoms: 10 min	13 items	Not reported	, etc.
PPRS (41)	1998	Israel	5–10 min	6 items	0.760-0.800	Hebrew, Spanish, etc.
NPI-Q (45)	1997	USA	10–15 min	3 dimensions, 12 items	0.851	Chinese, Spanish, Japanese, Dutch, Portuguese, etc.
MDS-NMS (49)	2020	UK	15–20 min	13 dimensions, 52 items	0.660	None

### 4.2 Comparative analysis of the clinical applications and practical utility

The evaluation of neuropsychiatric symptoms in Chinese patients with PD currently employs both general psychiatric rating scales (including PANSS, NPI-Q, and BPRS) and disease-specific instruments (such as MDS-UPDRS and SAPS-PD). Among these assessment tools, PANSS and BPRS are predominantly utilized for quantifying the severity of psychotic symptoms, while NPI-Q emphasizes caregiver-reported neuropsychiatric manifestations. Although MDS-UPDRS has gained widespread adoption in clinical screening due to its comprehensive nature, it demonstrates limited specificity for distinct psychotic subtypes. The comparative features of these instruments are summarized in **Table 2**. The eSAPS-PD shows particular efficacy in detecting minor phenomena (e.g., illusions), albeit requiring administration by trained professionals. While PPRS and PPQ facilitate rapid clinical assessments, their symptom coverage remains relatively restricted. PsycH-Q, as an emerging self-report instrument, exhibits high sensitivity (Cronbach’s alpha=0.900) but awaits validation in Chinese populations. The GAS, specifically developed for Chinese patients with PD, focuses on apathy assessment but necessitates complementary scales for comprehensive neuropsychiatric evaluation. General psychiatric scales remain widely used in clinical practice despite their inherent limitations in symptom specificity. In contrast, disease-specific tools like eSAPS-PD offer enhanced clinical relevance. Future research should prioritize rigorous psychometric validation of these instruments, with particular emphasis on their cross-cultural applicability and implementation in diverse clinical settings across China. This approach will facilitate more accurate characterization of neuropsychiatric symptoms in Chinese PD populations and support the development of targeted therapeutic interventions.

**Table 2:** Comparison of key properties of Parkinson's disease psychosis assessment tools.

Assessment Tool	Assessor	Recommended Disease Stage	Symptom Sensitivity/Specificity	Suitability for Cognitive Impairment	Cultural Validity in Chinese Population	Clinical utility
MDS-UPDRS Part 1A (16)	Clinicians	Early-stage	Only 6 items; low sensitivity; may miss non-hallucinatory psychosis	requires self-report; mild cognitive impairment may affect validity	Moderate reliability (Cronbach’s alpha = 0.734); lacks independent validation	Suitable for rapid screening only(less than 5 min); should be combined with other tools
PPQ (19)	Clinicians/Nurses	Early-stage	Focuses on medication-related symptoms; lacks PDP specificity	Relies on patient self-report; for basic screening only	No Chinese version available	Quick (7 min);suitable for baseline use; requires validation
SEND-PD (21)	Professionally administered	Mid-to-late-stage	Covers wide neuropsychiatric symptoms; emotion/apathy well rated; impulse control less reliable	Suitable for moderate cognitive impairment	No Chinese version available	Quick (5–10 min); suitable for outpatient or initial screening
PsycH-Q (24)	Patient self-report	Early-stage	High for frequency and severity of hallucinations and psychotic symptoms, but insensitive to negative symptoms	Suitable only for individuals without cognitive impairment	No Chinese version available	Clinically useful for efficacy evaluation and disease monitoring
GAS (25)	Patient self-report	Mid-to-late-stage	Focuses on apathy, culturally sensitive to social motivation in Chinese population; lacks psychotic symptom items	Not suitable for moderate/severe cognitive impairment	Locally developed	Time-consuming (10–15 min); better for in-depth evaluation or research
UM-PDHQ (18)	Clinicians	Mid-to-late-stage	Targets hallucinations; lacks robust validation; concerns over specificity	Requires third-party observation for severe impairment	No Chinese version available	Moderate length (10–15 min); limited applicability in general practice
TUHARS (26)	Clinicians	Early-stage	High reliability; only 7 items; may miss complex symptoms; sensitive to early PD psychosis	Suitable for mild/moderate impairment	Suitable for mild/moderate impairment	Efficient (5 min); suitable for quick screening, requires trained raters
PANSS (28)	Psychiatrists	advanced stage/Complex cases	Designed for schizophrenia; limited in detecting PDP-specific features	Complex and time-consuming (15 min) in cognitively impaired; better for cognitively normal	Good validity of Chinese version, but some items may require cultural adaptation	Low efficiency for routine screening; more suitable for research
BPRS (34)	Clinicians	advanced stage/Complex cases	Sensitive to positive symptoms; not PDP-specific. Risk of confusion between motor and psychotic features	Requires patient cooperation; less suitable for patients with severe cognitive impairment	High reliability in Chinese version, but items like “hostility” may need cultural contextualization	Time-consuming (30 min); better for research or severe cases
SAPS-PD (38)	Clinicians	advanced stage/Complex cases	Focused on positive symptoms (e.g., hallucinations); sensitive to minor phenomena but may overlook delusions	Not suitable for patients with severe cognitive impairment	Chinese version available, but lacks local psychometric data	Time-consuming (more than 30 min); mainly used in research
eSAPS-PD (40)	Clinicians	Early-stage	High sensitivity for PDP symptoms, especially minor hallucinations; specificity not quantified	Applicable to mild-to-moderate cognitive impairment	Psychometrically robust; validated in China	Effective for early detection, risk stratification, and longitudinal treatment monitoring
PPRS (41)	Clinicians	Mid-to-late-stage	Applicable to diagnosed PDP; poor for screening. Emphasizes hallucinations; delusions under assessed	Applicable for assessing patients with cognitive impairment	No Chinese version available	Suitable for quick screening (5 min); limited applicability
NPI-Q (45)	caregivers	Mid-to-late-stage	Covers core symptoms (hallucinations, delusions), but lacks sensitivity to minor phenomena	One of the best options for cognitively impaired patients	Chinese version available; some items (e.g., elation) may be culturally underestimated	Fast (10 min); suitable for outpatient screening, but subject to caregiver bias
MDS-NMS (49)	Clinicians/Nurses	Mid-to-late-stage	Covers mild–moderate psychiatric symptoms; limited focus on psychosis; lacks specificity	Applicable for mild to moderate cognitive impairment	No validated Chinese version; cultural adaptability unclear	time-consuming (20 min);Suitable for comprehensive assessments

### 4.3 Implementation challenges and research gaps in China

Cultural differences represent a key barrier to tool applicability. Symptom descriptions in Westerndeveloped scales often do not align with the typical presentations observed in Chinese patients. For example, “jealous delusions”, commonly noted in Western contexts, are less prevalent among Chinese individuals, who more frequently exhibit “persecutory” or “referential” delusions. Zhang et al (6) demonstrated that cultural context significantly influences the expression and interpretation of minor hallucinations in Chinese populations. These findings highlight the urgent need for culturally sensitive adaptation and validation of existing instruments. In terms of validation, many internationally developed tools lack Chinese versions or have not been validated in PDP populations. The PPQ and SEND-PD have not yet been translated into Chinese. The Chinese version of the NPI-Q has been primarily validated in dementia cohorts rather than in PDP populations. While the eSAPS-PD has been applied in a Chinese sample, large-scale psychometric validation remains lacking. Consequently, China still lacks a comprehensive PDP assessment system that is both culturally appropriate and methodologically rigorous. The implementation of clinical practices is further complicated by structural limitations. Tools such as PANSS, SAPS-PD, and BPRS require trained psychiatric professionals, making them impractical in most primary or community-level healthcare settings. Additionally, China’s historical one-child policy has led to caregiver shortages in many households, increasing the burden on individual caregivers and limiting the feasibility and reliability of informant-based tools like NPI-Q and self-report scales such as GAS. Furthermore, even tools that are widely adopted in clinical practice, such as the Chinese version of the MDS-UPDRS, have not been systematically validated for use in PDP. Current research in China is still at an early stage. Most studies are cross-sectional, single-center, and based on small samples. Locally developed, PDP-specific tools remain scarce, and few studies incorporate long-term follow-up or multicenter validation. To address these gaps, future research should prioritize (1) systematic validation of existing tools in diverse Chinese cohorts, (2) development of digital assessment platforms to increase accessibility, (3) simplification of tools for use in low-resource settings, and (4) implementation of structured training programs for clinical raters. Addressing these challenges is critical for establishing a standardized, scalable, and culturally valid framework for PDP assessment in China.

## 5 Device-added therapies of the patient with psychosis Parkinson’s disease

The use of device-aided therapies (DATs), such as DBS, is becoming increasingly common in advanced PD. However, PDP remains a critical concern when considering these interventions. Among DATs, DBS—particularly targeting the subthalamic nucleus (STN)—has been associated with an increased risk of exacerbating psychotic symptoms (50). Therefore, active PDP is typically regarded as a contraindication to DBS, and such patients should undergo comprehensive neuropsychiatric assessment before surgical consideration. In selected cases, stimulation of the globus pallidus interna (GPi) may present a more neuropsychiatrically favorable alternative (51). In contrast, LCIG is considered a relatively safer option for patients with well-managed PDP, particularly as it enables simplification of oral dopaminergic regimens, which may themselves contribute to psychosis (52). However, close monitoring is still essential to detect any worsening of neuropsychiatric symptoms. CSAI, while effective for motor fluctuations, may aggravate hallucinations or delusions (52), especially in patients with a history of PDP, and should be used cautiously. For patients with PDP requiring DATs, pre-treatment with atypical antipsychotic agents, such as pimavanserin or low-dose quetiapine, may help to stabilize psychiatric symptoms and create a window for DAT implementation (53). These considerations highlight the need for individualized therapeutic strategies and multidisciplinary evaluation in the context of neuropsychiatric comorbidities in PD.

## 6 Conclusion

This paper provides an overview of the assessment tools for PDP. Currently, research on PDP in China is relatively underdeveloped, and the available assessment tools mainly rely on generalized neuropsychiatric scales, which lack specificity. Therefore, future research should focus on developing and refining specialized PDP assessment tools tailored to the needs of the Chinese population. The aim is to enable early detection and comprehensive evaluation of PDP. This will support personalized treatment planning, reduce caregiver burden, and ultimately improve patients’ quality of life.
